# Human Umbilical Cord Mesenchymal Stem Cells Inhibit the Progression of Osteoarthritis by Suppressing NLRP3-Mediated Synovial Inflammation in the Early Stages of the Disease

**DOI:** 10.1155/sci/7558817

**Published:** 2025-08-30

**Authors:** Yu Li, Yu Ouyang, Ruibo Lang, Jing He, Shuo Zheng, Chunchun Ao, Yijia Jiang, Huan Xiao, Mao Li, Changyong Li, Dongcheng Wu

**Affiliations:** ^1^R&D Center, Wuhan Hamilton Biotechnology Co. Ltd, Wuhan, Hubei, China; ^2^School of Public Health, Hubei University of Medicine, Shiyan, Hubei, China; ^3^Department of Biochemistry and Molecular Biology, Wuhan University School of Basic Medical Sciences, Wuhan, Hubei, China; ^4^School of Life Sciences, Hubei University, Wuhan, Hubei, China; ^5^Department of Physiology, Wuhan University School of Basic Medical Sciences, Wuhan, Hubei, China; ^6^Xianning Medical College, Hubei University of Science and Technology, Xianning, Hubei, China; ^7^R&D Center, Guangzhou Hamilton Biotechnology Co. Ltd, Guangzhou, Guangdong, China

## Abstract

Osteoarthritis (OA) is the leading joint disease that causes joint pain and disability. Despite increasing progress regarding the therapeutic potential of human umbilical cord mesenchymal stem cells (UC-MSCs) for OA, effective strategies for the treatment of OA using UC-MSCs have not yet been developed in clinical practice. Our present study has proven that the early stage in OA rats is the main development stage of nod-like receptor heat protein domain protein 3 (NLRP3)-mediated synovial inflammation. The middle stage in OA rats is the main development stage of chondrocyte apoptosis. The late stage in OA rats is the main development stage of synovial fibrosis. The treatment of UC-MSCs in the early and middle stages of OA significantly reduced cartilage damage in rats, and improved the pathological structure of the knee joint. In comparison, UC-MSCs effectively reduced chondrocyte apoptosis in the early and middle stages of OA rats, but they only effectively reduced NLRP3-mediated synovial inflammation in the early stages of OA rats. Experiments in vitro showed that UC-MSCs could inhibit NLRP3-mediated pyroptosis of rat primary synovial cells (Rat-scs). In conclusion, our findings suggest that UC-MSCs exert therapeutic effects on OA, at least in part, through inhibiting NLRP3-mediated synovial inflammation in the early stage of OA.

## 1. Introduction

Osteoarthritis (OA) is a kind of aseptic inflammation characterized by the degeneration of articular cartilage, accompanied by synovial inflammation, subchondral sclerosis, and osteophyte formation. OA is the first joint disease that causes joint pain and disability, and the number of cases is increasing year by year. According to data and statistics reported by the World Health Organization, approximately 528 million people worldwide had OA in 2019, an increase of 113% since 1990 [1]. The knee is the most commonly affected joint, affecting 365 million people [1]. General conventional treatment can only relieve pain [2, 3], which usually leads to joint replacement in patients with end-stage OA.

Synovial inflammation is an important pathological process of OA and a key factor in promoting the pathological progression of OA and causing pain [4]. When OA occurs, synovial cells in the joints secrete proinflammatory factors and mediators such as matrix metalloproteinases (MMP), which enhance the catabolism of chondrocytes, transform macrophages into proinflammatory phenotypes, and lead to the degradation of the extracellular matrix. The proinflammatory factors secreted by inflammatory macrophages will accelerate the progression of inflammation, and tiny fragments of the extracellular matrix will lead to damage associated molecular patterns (DAMPs), further exacerbating OA [4]. Studies have shown that exosomes derived from inflammatory synovial cells can enhance macrophage glycolysis by upregulating HIF1A-mediated transcription of glycolysis-related genes, promote the transformation of macrophages in OA to the M1 phenotype, and aggravate the progression of OA [5]. Magnetic resonance imaging results of OA show that synovial inflammation is distributed in the patella, ligaments, and other parts of the joints of OA patients [6].

nod-like receptor heat protein domain protein 3 (NLRP3) can initiate the assembly of inflammasomes, induce the secretion of Interleukin (IL)-1β and IL-18, and lead to inflammatory cell death, a process known as pyroptosis [7]. Under normal circumstances, when invaded by bacteria, fungi, viruses, et cetera, inflammasomes will activate the immune system to protect the body, and inflammatory factors at this time can eliminate foreign invaders [8]. Under pathological conditions, when the regulation of inflammasomes is out of control, a large number of inflammatory factors will be produced to damage the body [9]. Studies have shown that NLRP3-mediated pyroptosis can activate toll-like receptors and NF-κB signaling pathways, and participate in OA and synovial inflammation [10]. Through experiments on clinical patients, some studies believe that IL-1β involved in cartilage decomposition in OA is not produced by chondrocytes, but by synovial tissue [11]. By comparing the synovial tissue of normal bodies and OA patients, it was found that the NLRP3 protein in the synovial tissue of OA patients was significantly increased [12]. Recent studies have found that ALPK1 accelerates the onset of OA by activating NLRP3 signal transduction [13]. Therefore, NLRP3-mediated pyroptosis may participate in the pathological process of OA through synovial inflammation and play an important role. NLRP3 may be an important biomarker for the diagnosis and monitoring of OA [14].

In recent years, the treatment of OA with mesenchymal stem cells (MSCs) has become a research hotspot. MSCs are a type of multipotent stem cells that exist in connective tissue and the stroma of various organs. They have secretory, immunomodulatory, and homing properties and can differentiate into adipocytes, chondrocytes, and osteoblasts in vitro [15]. In addition, the proteins and microRNAs secreted by MSCs and their extracellular vesicles (EVs) are involved in multiple biochemical processes such as communication, inflammation, tissue repair, and metabolism. These functions are the focus of attention in the field of regeneration and tissue repair [16]. In terms of the source of MSCs, umbilical cord MSCs (UC-MSCs) have good characteristics such as rich tissue sources, strong expansion ability, and low immunogenicity. In recent studies, it has been proposed that UC-MSCs exosomes (UC-MSC-EXOs) can be targeted to chondrocytes to treat OA by restoring senescent chondrocytes [17]. Several studies have reported that enriched engineered MSC-EXOs can enhance their therapeutic effects on OA [18–20]. In studies related to NLRP3, it was shown that MicroRNA-223 from UC-MSC-EXOs can directly bind to the 3′UTR of NLRP3 mRNA to inhibit chondrocyte pyroptosis, thereby, maintaining chondrocyte homeostasis and alleviating OA [21].

However, there are still some problems in using MSCs to treat OA in clinic. The pathological mechanism of OA is complex. Cartilage damage is the direct cause of joint function loss, but apoptosis, inflammation and fibrosis play an important role in promoting the progression of OA. The microenvironment in the joint affects all cell types [22, 23]. Mitochondrial dysfunction, DAMPs, cytokines, metabolites, and crystals in the synovium can all lead to the transmission of OA-related signals between chondrocytes, synovial cells, and immune cells in the joint, and then promoting the progression of OA [4]. The therapeutic mechanism of MSCs has not yet been clearly explored. Although MSCs coordinate local and systemic innate and adaptive immune responses by releasing various mediators including immunosuppressive molecules, growth factors, exosomes, chemokines, complement components, and various metabolites [24], different diseases require different treatment strategies. Even if the same type of MSCs is used for treatment, different treatment plans are required for OA patients at different stages of the disease. In different disease stages, as a treatment object, which cell types are appropriate? In the early stage, the best treatment time of OA, can effective therapeutic strategy be formed? These issues are urgent to be solved in clinical practice.

In summary, the efficacy of MSCs in treating OA is increasingly recognized, but there are still many urgent problems to be solved in clinical application. Moreover, the target cells of this type of research are all chondrocytes, and the purpose is to inhibit chondrocyte inflammation and cartilage degradation. However, only a limited number of studies have investigated whether MSCs can alleviate OA by inhibiting synovial inflammation and NLRP3-mediated synovial cell pyroptosis, which are critical pathological manifestations and significant cell signaling pathways in OA [4]. There is no research report indicating which stages of OA are appropriate for using MSCs as a treatment strategy for synovial inflammation.

This study used sodium iodoacetate-induced OA rats as a model to explore the pathological processes of synovial inflammation, chondrocyte apoptosis, and synovial fibrosis in the early, middle, and late stages of OA. It was found that the early stage of OA is the main development stage of synovial inflammation, the middle stage of OA is the main development stage of chondrocyte apoptosis, and the late stage of OA is the main development stage of synovial fibrosis. NLRP3 protein is significantly up-regulated in the synovial tissue during the early stages of the disease. Through the treatment of OA rats with UC-MSCs at all stages of the disease course, it was found that UC-MSCs treatment in the early and middle stages reduced cartilage damage and inhibited chondrocyte apoptosis. UC-MSCs significantly inhibited NLRP3-mediated synovial cell pyroptosis in the early stages of OA rats, and also inhibited the expression of NLRP3 protein in rat synovial cells in vitro. The above experimental results illustrate the therapeutic strategy of UC-MSCs in the early stage of OA rats, which is mainly to inhibit NLRP3-mediated synovial inflammation.

## 2. Materials and Methods

### 2.1. Animal Experiment

Purchase 200–250 g specific pathogen free (SPF) grade Sprague–Dawley male rats at the Bestcell Model Biological Center (Wuhan, China). The experimental rats were fed under conditions of 45%–55% humidity, 20 ± 2°C temperature, normal feeding, and water through 12 h light and dark cycle. The animal rats are raised in accordance with the Regulations on the Management of Laboratory Animals and the Guidelines on the Management and Use of Laboratory Animals, approved by the Laboratory Animal Welfare and Ethics Management Committee of the Bestcell Model Biology Center, and the project number is (No. 2023-05-17A).

Rats were randomly divided into two groups: OA group (*n* = 30) and control group (*n* = 5). According to the sodium iodoacetate (MIA) dosage of 1.5 mg/50 μL per 200 g body weight, MIA solution was injected into the knee cavity of the rats in the OA group, and equal volume pure water was injected into the knee cavity of the control group, and the administration day was 0. The injection method for MIA and UC-MSC involves penetrating the joint cavity with a syringe and administering the liquid.

After 3 days, the rats in the OA group were randomly divided into six groups: the pathological group in the first week of OA (OA-1W, *n* = 5); the pathological group at the second week of OA (OA-2W, *n* = 5); OA pathology at week 3 (OA-3W, *n* = 5); UC-MSCs treatment group at week 1 (MSC-Early, *n* = 5); UC-MSCs treatment group at week 2 (MSC-Mid, *n* = 5); UC-MSCs treatment group at week 3 (MSC-Late, *n* = 5). On the same day, UC-MSCs in the knee cavity of MSC-Early group were injected with 2.5 × 10^5^/50 μL normal saline.

At 1 week and 2 weeks from day 0, rats in OA-1W group and OA-2W group were given excessive isoflurane inhalation, and the knee joint and part of the synovial tissue were removed. The joint was soaked in 4% paraformaldehyde and fixed for use. The synovium was frozen in the ultra-low temperature refrigerator at −80°C after quick freezing with liquid nitrogen.

The MSC-Mid group and the MSC-Late group were treated with US-MSCs 1 week and 2 weeks after the treatment of the MSC-Early group, respectively, and 2.5 × 10^5^/50 μL of normal saline was injected into the knee cavity of the rats.

At 3 weeks from day 0, the rats in the sacrificed control group, OA-3W group, MSC-Early group, MSC-Mid group, and MSC-Late group were given excessive isoflurane inhalation, and the knee joint and part of the synovial tissue of the joint cavity were removed. The joint was soaked in 4% paraformaldehyde and fixed for use. The synovium was frozen in the ultra-low temperature refrigerator at −80°C after quick freezing with liquid nitrogen.

Detailed information about animal experiments can be viewed in ARRIVE checklist of the Supporting Information. For detailed animal experimental procedures, please refer to Figure S1.

### 2.2. Cell Culture and Treatment

The UC-MSCs were freshly isolated from human umbilical cord tissue after deliveries in Renmin Hospital of Wuhan University (Permit Number: WDRY2019-G001). UC-MSCs were isolated to meet the characteristics of MSCs using methods described previously [25]. UC-MSCs were stored in serum-free stem cell culture medium with additives (NC0103, YOCON, China) and cultured at 37°C with 5% CO_2_. The cell-free culture supernatant of UC-MSCs was collected and then passed through 0.22 μm sterile filter (BS-PES-22, BioSharp, China). The primary human UC-MSCs were harvested when the cell confluence reached 80% and then expanded by regular subculture. The human UC-MSCs at Passage 5 (P5) were harvested as working cells and used in this study.

At P5, the UC-MSCs were characterized by flow cytometry using the following fluorescently labeled antibodies (BioLegend, USA): anti-human CD73 (cat. No. 344,016), CD90 (cat. no. 328,110), CD105 (cat. no. 323,204), CD34 (cat. no. 343,604), CD44 (cat. no. 103,007), CD45 (cat. no. 304,008), CD19 (cat. no. 302,208), and HLA-DR (cat. no. 307,606) antibodies as described previously [26]. Figures S2 and S3 present the characterization of unstained negative controls for MSCs as well as serum-containing versus serum-free culture controls. Cell phenotype was determined using a flow cytometer (CytoFLEX S, Beckman Colter, USA). For multipotent differentiation potential, human UC-MSCs were maintained in adipogenic (cat. no. A10070), chondrogenic (cat. no. A10071), and osteogenic (cat. no. A10072) differentiation media, respectively, following the manufacturer's instruction (Gibco, USA). Successful inductions of adipogenesis, osteogenesis, and chondrogenesis were confirmed by staining with Oil Red O, Alizarin Red, and Alcian Blue (Gibco).

The joint tissue of rats was taken, the surface tissue muscle was removed, and the synovial tissue (semi-transparent film) was stripped along the side of the joint cavity with surgical scissors. After washing PBS for time times, add ten times the volume of 0.25% pancreatic enzyme (25,200–072, Gibco, USA) and digest at 37°C for half an hour to 1 h. Pancreatic enzyme was absorbed and washed twice with PBS, then DMEM (C11885500BT, Gibco, USA) medium containing 0.02% collagenase II (17101015, Gibco, USA) and penicillin and streptomycin (15140122, Gibco, USA) was added and digested at 37°C overnight. On the second day, the digested liquid was blown several times, filtered, and cultured with high-sugar medium containing 10% FBS (10,099-141c, Gibco, USA) and DMEM medium with penicillin and streptomycin, 48 h after cell adhesion, 3 days after cell fluid replacement.

Rat primary synovial cells (Rat-scs) were inoculated with six-well plates at 1 × 105 per well, adhered to the wall for 24 h, and the cell confluence reached 60% after another 24 h, and were replaced with serum-free DMEM high-sugar medium and starved for 6 h. After incubation, 10% FBS-DMEM high-glucose medium was added to the treatment group, and 1000 ng/mL LPS (L3129-10MG, Merck, China) was added to both groups for overnight stimulation (18 h). On the second day, both groups were stimulated with 5 mmol/L ATP for 1 h, cell samples were collected, and supernatant was frozen at −80°C for use.

### 2.3. Histology, Immunofluorescence, and Immunohistochemistry

The knee sections of rats were fixed with 4% paraformaldehyde and embedded in paraffin to prepare 5 μm sections. The slices are decarbonized in xylene and rehydrated in water through a series of graded concentrations of ethanol. Hematoxylin and eosin (H&E) staining were used to evaluate the tissue morphology of the paraffin sections, and safranin-O staining was used to detect the injury status of articular cartilage. The morphological and pathological changes of the joints were recorded under an optical microscope (Olympus, Japan) and histologically assessed using Pelletier [27] and OARSI [28] scores.

After deparaffinization and rehydration, sections were microwaved in citrate buffer for antigen retrieval. Samples were incubated with 3% hydrogen peroxide at room temperature in the dark to block endogenous peroxidase activity. Samples were then blocked with 3% BSA for 30 min to prevent nonspecific binding. Subsequently, the sections were stained with anti-NLRP3 (1:300, GB114320, Servicebio, China), anti-Gasdermin D (GSDMD) (1:300, GB114198, Servicebio, China), anti-IL-1β (1:300, GB11113, Servicebio, China), anti-MMP13 (1:300, GB11247, Servicebio, China) and anti-(type Ⅰ collagen) (COL-1) (1:1000, GB11022, Servicebio, China) at 4°C overnight. Secondary antibodies conjugated with HRP were applied. DAB (G1211) was used for staining, followed by light counterstaining with hematoxylin. Finally, the slides were dehydrated and mounted, and tissue staining was observed under a microscope (E100, DS-U3Nikon). Chondrocytes apoptosis was assessed by terminal deoxynucleotidyl transferase-mediated dUTP Nick-End Labeling (TUNEL) staining (GDP1042, Servicebio, China). Slides were analyzed under a fluorescence microscope (Eclipse Ci-E, Nikon, Japan), and images were processed and analyzed using ImageJ.

### 2.4. Quantitative Real-Time Polymerase Chain Reaction (qRT-PCR)

Total RNA was isolated from rat synovial cells with TRIzol reagent (9109 Takara, Japan). Using PerfectStart Uni RT&qPCR Kit (AUQ-01, Transgen, China), about 1 μL RNA of each sample is used for cDNA synthesis. qRT-PCR reaction using PerfectStart Uni RT&qPCR Kit (AUQ-01, Transgen, China) on CFX Connect Optics Module (Bio-Rad, USA). The relative expression levels of target genes were calculated using the 2^−ΔΔCT^ method. The primer sequences are shown in Table S1. The reverse transcription system and qPCR system are shown in Table S2 and Table S3, respectively. The qPCR program settings are presented in Table S4. The quality parameters of RNA can be found in Table S5.

### 2.5. Western Blotting

The total protein was extracted by RIPA extraction buffer (P0013B, Beyotime, China) and the protein concentration was determined by BCA detection kit (P0009, Beyotime, China). The protein extract was then separated with 8% or 10% SDS–PAGE gel and transferred to PVDF membrane (ISEQ00010, Millipore, USA). After that, the membrane was blocked with 5% milk at room temperature for 1 h.

The primary antibody against NLRP3 (1:1000, NBP2-12446, NOVUS, USA) was purchased from NOVUS, GSDMD (1:1000, SC-393656, SANTA CRUZ, USA) actin was purchased from SANTA CRUZ, Caspase1 (1:1000, 22,915-1-AP, Proteintech, USA) and β-actin (1:5000, 66,009-1-Ig, Proteintech, USA) were purchased from Proteintech. After incubation with the secondary antibody, antibody binding was detected using chemiluminescence with an ECL detection kit. The original unprocessed western blot images can be viewed in Figure S4 and Figure S5.

### 2.6. Enzyme-Linked Immunosorbent Assay (ELISA)

The concentrations of IL-1β in rat snovial cells were measured using the ELISA kit (E-EL-R0012c, Elabscience, China) according to the manufacturer's instructions. Cytokine levels were presented by cytokine concentration/albumin concentration.

### 2.7. Statistical Analysis

All data are expressed as mean ± standard deviation (SD). Intergroup differences were evaluated using an unpaired Student's *t*-test for comparisons between two samples, or analysis of variance (ANOVA) for multiple comparisons. Data were first assessed for normality using the Shapiro–Wilk test, followed by evaluation of variance homogeneity with the Brown–Forsythe test. Statistical analyses were conducted utilizing GraphPad Prism software. A *p*-value of less than 0.05 was considered indicative of statistical significance.

## 3. Results

### 3.1. Characterization of UC-MSCs

**Figure 1:**
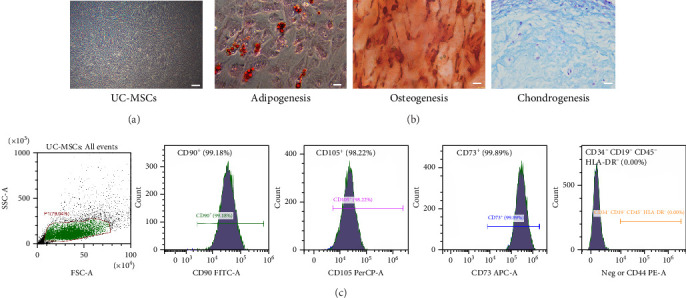
Characterization of UC-MSCs. (A) Under microscope, the growth morphology of UC-MSCs for 72 h was spindle type. Scale bar: 100 μm. (B) UC-MSCs induced differentiation and staining in vitro. The adipogenic, osteogenic, and chondrogenic induction of UC-MSCs were detected by oil red O, alizarin red, and Alcian blue staining. Scale bar: 80 μm. (C) CD90, C105, and CD73 were positive on the surface of UC-MSCs. The negative mixture (CD34, CD19, CD45, and HLA-DR) on the surface of UC-MSCs showed negative co-staining.

**Figure 2:**
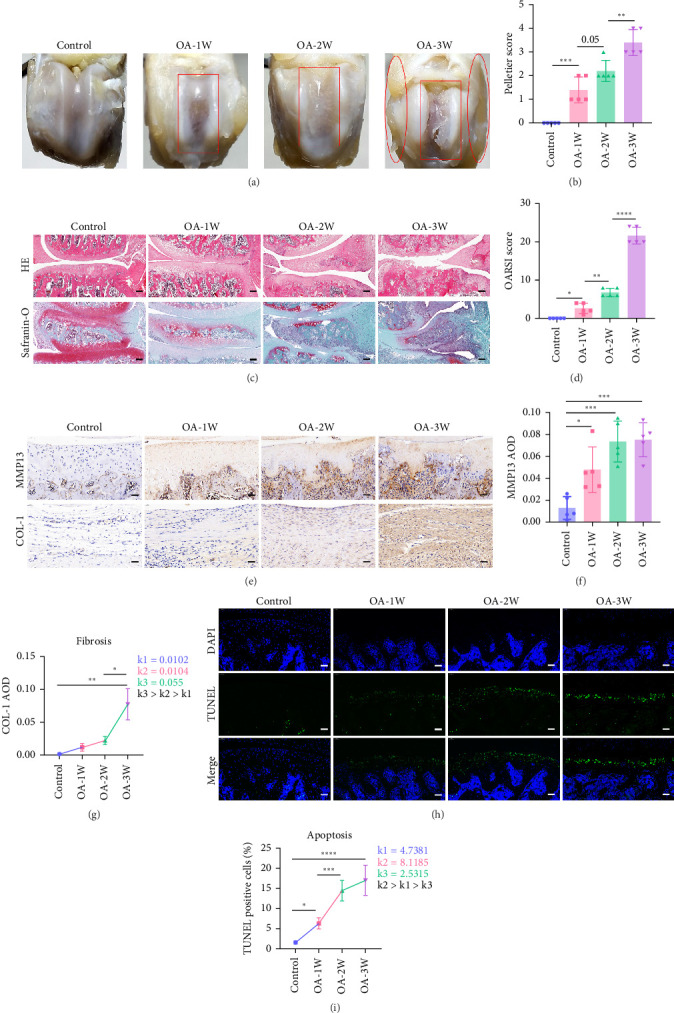
The middle and late stages of OA rats are the main development stages of chondrocyte apoptosis and synovial fibrosis. (A) Changes in the surface morphology of rat knee cartilage after intra-articular injection of 1.5 mg/200 g MIA on day 0 for 3 consecutive weeks (*n* = 5 for each group). The rectangular regions indicate cartilage damage, while the elliptical regions denote synovial hyperplasia. (B) Pelletier scores of each group (*n* = 5 for each group). (C) Representative images of H&E staining and safranin-O staining (*n* = 5 for each group). Scale bar: 200 μm. (D) OARSI scores of each group (*n* = 5 for each group). (E) Immunohistochemistry staining of cartilage MMP13 and synovial COL-1 (*n* = 5 for each group). Scale bar: 50 μm. (F) The average optical density (AOD) of cartilage MMP13 in each group (*n* = 5 for each group). (G) The weekly slope *k* of COL-1 AOD in OA rats during each week of 3-week pathological process alone. (H) Chondrocyte TUNEL staining of rats in each group. Scale bar: 50 μm. (I) The weekly slope *k* of TUNEL-positive cell rate of chondrocytes in OA rats during each week of 3-week pathological process alone. Data are presented as mean ± SD of individuals included in each group. *⁣*^*∗*^*p* < 0.05, *⁣*^*∗∗*^*p* < 0.01, *⁣*^*∗∗∗*^*p* < 0.001, *⁣*^*∗∗∗∗*^*p* < 0.0001.

Under the microscope, UC-MSCs was fibroblast-like spindle, and the cells grew in vortex and arranged in parallel (Figure 1A). UC-MSCs was induced by adipogenesis, osteogenesis, and chondrogenesis, followed by histological staining (Figure 2B). Flow cytometry showed that the expression of CD90, C105, and CD73 on the surface of UC-MSCs cells was strongly positive (>98%), while the expression of CD34, CD19, CD45, and HLA-DR was negative (<1%) (Figure 1C). The results show that the UC-MSCs used in the experiment can adhere to the wall and show typical MSC morphology, have strong three-line differentiation ability and MSC surface markers, and meet the identification standards of the International Society for Cellular Therapy (ISCT) [29].

### 3.2. The Middle and Late Stages of OA Rats Are the Main Development Stages of Chondrocyte Apoptosis and Synovial Fibrosis

In order to explore the therapeutic strategy of UC-MSCSs for OA, it is essential to understand the pathological development of OA throughout the course of the disease. This study used an MIA-induced OA rat model and took samples continuously at the first week (OA-1W), second week (OA-2W), and third week (OA-3W) after modeling. The surface morphology of the knee cartilage showed (Figure 2A) that compared with the control group, the surface damage of the articular cartilage at different pathological time points gradually worsened over time, and the synovial tissue gradually proliferated. The rectangular regions indicate cartilage damage, while the elliptical regions denote synovial hyperplasia. The Pelletier score [30] related to knee cartilage damage was significantly different in every two adjacent weeks (Figure 2B). HE and safranin-O staining of joint sections (Figure 2C) revealed that the control group exhibited an intact knee cartilage structure with a uniform and orderly distribution of chondrocytes. The cartilage tissues of rats in the OA-1W, OA-2W, and OA-3W groups all showed varying degrees of wear. The cartilage edges of rats in the OA-1W group began to become uneven, the number of chondrocytes in the OA-2W group decreased, and the thickness of the cartilage layer became thinner. The damage and erosion of rats in the OA-3W group were severe. The red area representing the cartilage in safranin-O staining was significantly reduced or even disappeared, and the joint structure changed significantly (Figure 2C). The OARSI score [28] related to cartilage damage showed significant differences in the scores every two consecutive weeks (Figure 2D). MMP13 is an important marker of cartilage damage. During the development of OA, MMP13 secreted by chondrocytes leads to cartilage matrix degradation. The fragments of cartilage matrix in the joint tissue stimulate chondrocytes and synovial cells, leading to cell inflammation [31]. Immunohistochemistry was used to detect cartilage MMP13, and the results showed that cartilage MMP13 was significantly increased in the OA-1W, OA-2W, and OA-3W groups compared with the control group (Figure 2E,F). Cartilage damage, as the most important pathological manifestation of OA, directly reflects the pathological stage of OA rats. The Pelletier score and OARSI score indicate the establishment of the early, middle, and late stages of knee OA in rats. The MMP13 results show that the signaling pathway related to cartilage damage is activated from the early stage of the disease and continues to the late stage.

COL-1 is an important marker of fibrosis, indicating the occurrence and development of synovial hyperplasia. Immunohistochemistry was used to detect the expression of fibrosis signaling protein COL-1 in the synovial tissue of the rat knee joint (Figure 2E). The results showed (Figure 2G) that the synovial fibrosis rate was fastest in the OA-3W group, that is, in the late stage of the disease, and developed slowly in the early and middle stages of the disease, which was the same as the development trend of the synovial morphology of the rat knee joint (Figure 2A).

As the main cells in bone joints, chondrocytes are very important for the synthesis of cartilage matrix in joints and play an important role in the development of OA. Studies have shown that interference with OA-related signaling proteins in chondrocytes can significantly improve the progression of joint damage and reduce pain [32]. TUNEL staining (Figure 2H) showed that compared with the control group, the apoptosis of chondrocytes during the pathological process of rats was significantly increased in the OA-1W, OA-2W, and OA-3W groups, and increased with the development of pathological time (Figure 2I), and there was a significant difference between the apoptosis rates of the OA-1W group and the OA-2W group. Analysis of the chondrocyte apoptosis rate throughout the course of the disease showed (Figure 2I) that the chondrocyte apoptosis rate reached the fastest rate in the middle of the disease stage of OA.

### 3.3. The Early Stage of OA Rats is the Main Development Stage of NLRP3-Mediated Synovial Inflammation

**Figure 3:**
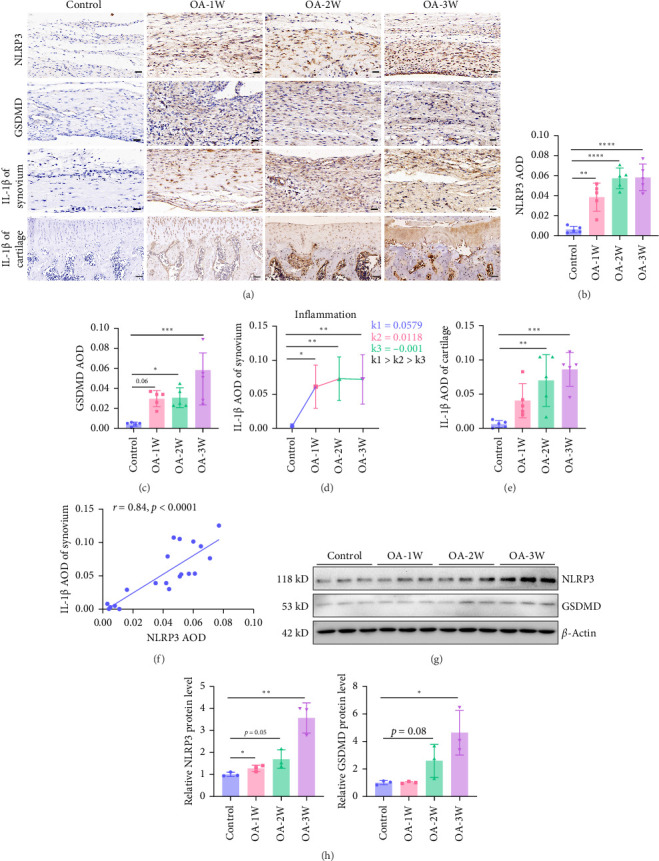
The early stage of OA rats is the main development stage of NLRP3-mediated synovial inflammation. (A) Immunohistochemistry staining of synovial NLRP3, GSDMD, IL-1β of synovium, and IL-1β of cartilage (*n* = 5 for each group). Scale bar: 50 μm. (B) The AOD of synovial NLRP3 in each group (*n* = 5 for each group). (C) The AOD of GSDMD in each group (*n* = 5 for each group). (D) The weekly slope *k* of IL-1β of synovium AOD in OA rats during each week of 3-week pathological process alone. (E) The AOD of IL-1β of cartilage in each group (*n* = 5 for each group). (F) GraphPad Prism 8.0.2 was used to analyze the correlation of NLRP3 AOD and IL-1β of synovium AOD in control, OA-1W, OA-2W, and OA-3W groups, respectively. (G) The protein levels of synovial NLRP3 and GSDMD were measured by western blot. (H) Relative protein levels of synovial NLRP3 (*n* = 3 for each group) and relative protein levels of synovial GSDMD (*n* = 3 for each group). Data are presented as mean ± SD of individuals included in each group. *⁣*^*∗*^*p* < 0.05, *⁣*^*∗∗*^*p* < 0.01, *⁣*^*∗∗∗*^*p* < 0.001, *⁣*^*∗∗∗∗*^*p* < 0.0001.

Synovial inflammation plays an important role in the development of OA. Synovial inflammation is involved in OA caused by aging, obesity, injury, and mechanical load, and NLRP3-mediated synovial cell pyroptosis is an important factor leading to inflammation [4]. NLRP3 is a marker of the initiation of cell pyroptosis signaling, and GSDMD is direct evidence of the formation of inflammasome complexes and cell pyroptosis, and pyroptotic cells release IL-1β. Immunohistochemistry detected the expression of pyroptosis signaling proteins NLRP3, GSDMD, IL-1β, and cartilage IL-1β in synovial tissue (Figure 3A). The results showed that compared with the control group, NLRP3, GSDMD, and IL-1β were significantly increased in the synovial tissue of rats in the OA-1W, OA-2W, and OA-3W groups (Figure 3). The expression of IL-1β in cartilage tissue was significantly increased in the OA-2W and OA-3W groups, and there was an increasing trend in OA-1W with no significant difference (Figure 3E), which indicates that cartilage inflammation lags behind the synovium relatively and cartilage inflammation begins to develop rapidly in the middle of the disease stages. Analysis of IL-1β expression changes in synovial tissue throughout the course of the disease (Figure 3D) shows that synovial inflammation develops fastest in the early stages of the disease, which is the main development stage of synovial inflammation. The correlation analysis between synovial IL-1β and NLRP3 further showed that NLRP3 is an important signaling molecule in mediating synovial inflammation (Figure 3F). The results of western blot (Figure 3G,H) further showed that NLRP3 and GSDMD were significantly increased in the synovial tissue of OA rats.

### 3.4. UC-MSCs Alleviate Cartilage Damage in Early and Middle Stages of OA Rats

**Figure 4:**
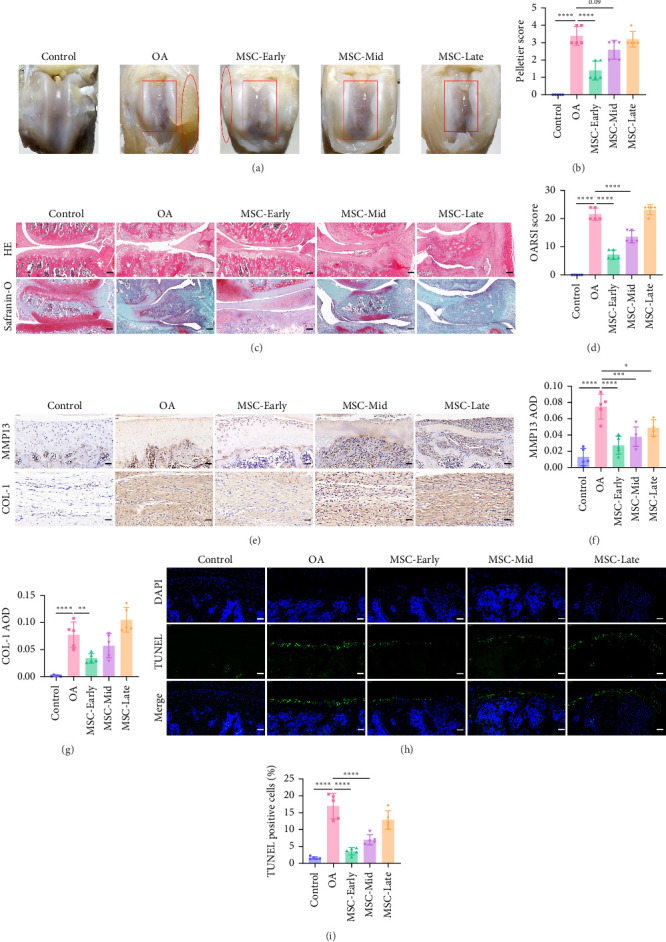
UC-MSCs alleviate cartilage damage in early and middle stages of OA rats. (A) Changes in the surface morphology of the rat knee cartilage after injection of 2.5 × 10^5^/50 μL UC-MSCs in PBS into OA rats at the 1^st^ week (MSC-Early), 2^nd^ week (MSC-Mid), and 3^rd^ week (MSC-Late) (*n* = 5 for each group). The rectangular regions indicate cartilage damage, while the elliptical regions denote synovial hyperplasia. (B) Pelletier scores of each group (*n* = 5 for each group). (C) Representative images of H&E staining and safranin-O staining (*n* = 5 for each group). Scale bar: 200 μm. (D) OARSI scores of each group (*n* = 5 for each group). (E) Immunohistochemistry staining of cartilage MMP13 and synovial COL-1 (*n* = 5 for each group). Scale bar: 50 μm. (F) The AOD of cartilage MMP13 in each group (*n* = 5 for each group). (G) The AOD of cartilage COL-1 in each group (*n* = 5 for each group). (H) Chondrocyte TUNEL staining of rats in each group. Scale bar: 50 μm. (I) The TUNEL-positive cell rate in each group (*n* = 5 for each group). Data are presented as mean ± SD of individuals included in each group. *⁣*^*∗*^*p* < 0.05, *⁣*^*∗∗*^*p* < 0.01, *⁣*^*∗∗∗*^*p* < 0.001, *⁣*^*∗∗∗∗*^*p* < 0.0001.

The above experiments showed that OA rats had different pathological characteristics in the early, middle, and late stages of the disease. In order to explore the difference in the efficacy of UC-MSCs on OA rats in the early, middle, and late stages of the disease, we used OA rats in week 3 as the model group, and the treatment group was divided into three groups. 2.5 × 10^5^/50 μL UC-MSC in PBS was injected intra-articularly at the early pathological stage of week 1 (MSC-Early), the middle pathological stage of week 2 (MSC-Mid), and the late pathological stage of week 3 (MSC-Late). The cartilage morphology photos showed that compared with the OA group, the articular cartilage surface injury in the MSC-Early group was significantly restored, and the synovial tissue proliferation was significantly reduced. The degree of recovery of articular cartilage surface injury in the MSC-Mid group was reduced. The therapeutic effect of the MSC-Late group on cartilage surface injury and synovial proliferation was poor (Figure 4A). Compared with OA, the Pelletier scores in MSC-Early group and MSC-Mid group showed a significant downward trend (Figure 4B). HE and safranin-O staining results showed that compared with the model group, the degree of cartilage tissue wear in the MSC-Early group was significantly reduced, the uneven cartilage edge was more restored, the number of chondrocytes increased, the thickness of the cartilage layer became thicker, and the red area of the cartilage increased (Figure 4C). The degree of cartilage tissue wear in the MSC-Mid group was reduced, the uneven cartilage edge was less restored, the erosion was weakened, and the red cartilage area increased slightly. No therapeutic effect was observed in the MSC-Late treatment group. Compared with the model group, the OARSI score gradually decreased significantly in the MSC-Early and MSC-Mid groups (Figure 4D), indicating that UC-MSCs had a significant effect on cartilage damage in the early and middle stages of the disease. The immunohistochemical results (Figure 4E–G) of MMP13 and COL-1 showed that UC-MSCs could reduce the expression of cartilage MMP13 in the early, middle, and late stages of the disease, and could only reduce the expression of synovial COL-1 in the early stage of the disease.

TUNEL staining results showed (Figure 4H,I) that the TUNEL-positive cell rate in the MSC-Early and MSC-Mid groups was significantly lower than that in the model group, while there was no significant change in the TUNEL-positive cell rate in the MSC-Late group, indicating that UC-MSCs can reduce chondrocyte apoptosis in OA rats in the early and middle stages of the disease. The above experiments show that UC-MSCs have a good therapeutic effect in the early and middle stages of OA rats and can reduce cartilage damage and chondrocyte apoptosis. Synovial fibrosis was alleviated by UC-MSCs in the early stage of OA rats.

### 3.5. UC-MSCs Inhibit NLRP3-Mediated Synovial Inflammation in the Early Stage of OA Rats

**Figure 5:**
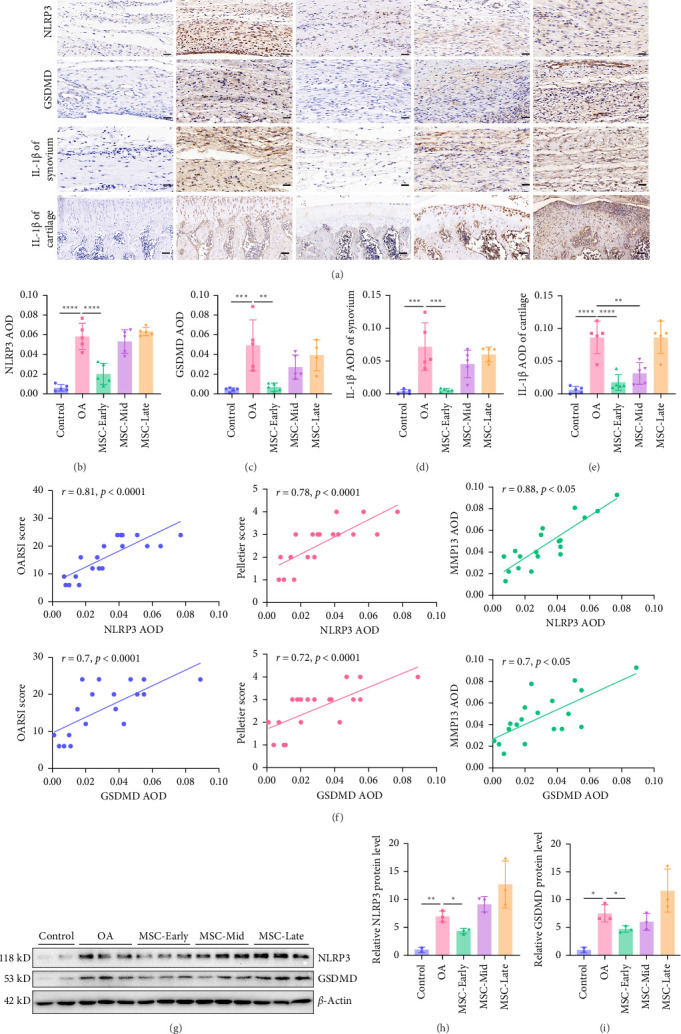
UC-MSCs alleviate NLRP3-mediated synovial inflammation in the early stage of OA rats. (A) Immunohistochemistry staining of synovial NLRP3, GSDMD, IL-1β of synovium, and IL-1β of cartilage (*n* = 5 for each group). Scale bar: 50 μm. (B) AOD of NLRP3 in each group (*n* = 5 for each group). (C) AOD of GSDMD in each group (*n* = 5 for each group). (D) AOD of IL-1β of synovium in each group (*n* = 5 for each group). (E) AOD of IL-1β of cartilage in each group (*n* = 5 for each group). (F) GraphPad Prism 8.0.2 was used to analyze the correlations of NLRP3 AOD and GSDMD AOD with OARSI score, Pelletier score, and MMP13 AOD were detected in OA, MSC-Early, MSC-Mid, and MSC-Late groups, respectively. (G) The protein levels of NLRP3 and GSDMD were measured by western blot (*n* = 3 for each group). (H) Relative protein levels of NLRP3 in each group (*n* = 3 for each group). (I) Relative protein levels of GSDMD in each group (*n* = 3 for each group). Data are presented as mean ± SD of individuals included in each group. *⁣*^*∗*^*p* < 0.05, *⁣*^*∗∗*^*p* < 0.01, *⁣*^*∗∗∗*^*p* < 0.001, *⁣*^*∗∗∗∗*^*p* < 0.0001.

From the previous experiments, we know that UC-MSCs has the best effect on cartilage damage in the early stages of OA rats, and the early stages of OA rats is the main development stage of NLRP3-mediated synovial inflammation. In order to explore the difference in the efficacy of UC-MSCs on NLRP3-mediated synovial inflammation and cartilage inflammation in the early, middle, and late stages of the disease, we used immunohistochemistry to detect synovial NLRP3, GSDMD, IL-1β, and cartilage IL-1β (Figure 5A). The results showed that UC-MSCs effectively inhibited the expression of synovial NLRP3, GSDMD and IL-1β in the early stages of the disease, while the expression of cartilage IL-1β was effectively inhibited in the early and middle stages of the disease (Figure 5B–E). This is similar to the treatment of chondrocyte apoptosis. In general, if treatment is given before or during the main stages of inflammation or apoptosis of chondrocytes, inflammation and apoptosis can be effectively inhibited. If the treatment time is after the main stages of inflammation or apoptosis, inflammation and apoptosis cannot be effectively inhibited. We speculate that after the main course of inflammation or apoptosis, the synovial inflammation worsens or the number of apoptotic chondrocytes is too large, and UC-MSCs cannot save the synovial cells or apoptotic chondrocytes in the tissue due to excessive inflammation, so the treatment is ineffective. Cartilage IL-1β only begins to be significantly expressed in the middle stage of OA rats, which is also the main development stage of chondrocyte apoptosis. Therefore, UC-MSCs can effectively inhibit the expression of IL-1β in cartilage tissue in the middle stage of the disease (Figure 5E).

During the early, middle, and late treatment of OA rats with UC-MSCs, NLRP3 and GSDMD were also positively correlated with cartilage damage-related indicators OARSI score, Pelletier score and MMP13, respectively (Figure 5F). This indicates that the attenuation of NLRP3-mediated synovial inflammation plays an important role in the treatment of OA cartilage damage by UC-MSCs. The reduction of NLRP3-mediated synovial inflammation by UC-MSCs is an important therapeutic direction for UC-MSCs in the treatment of OA. Western blot detected NLRP3 and GSDMD in synovial tissue. Compared with the OA group, the protein expressions of NLRP3 and GSDMD were significantly reduced in the early stages of treatment (Figure 5). It also once again demonstrated the good efficacy of UC-MSCs on NLRP3-mediated synovial inflammation in the early stages of OA rats. In summary, it indicates that UC-MSCs attenuate NLRP3-mediated synovial inflammation in the early stages of OA rats and can be used as a therapeutic strategy for OA treated with UC-MSCs.

### 3.6. UC-MSCs Inhibit NLRP3-Mediated Pyroptosis of Primary Synovial Cells of Rat In Vitro

**Figure 6:**
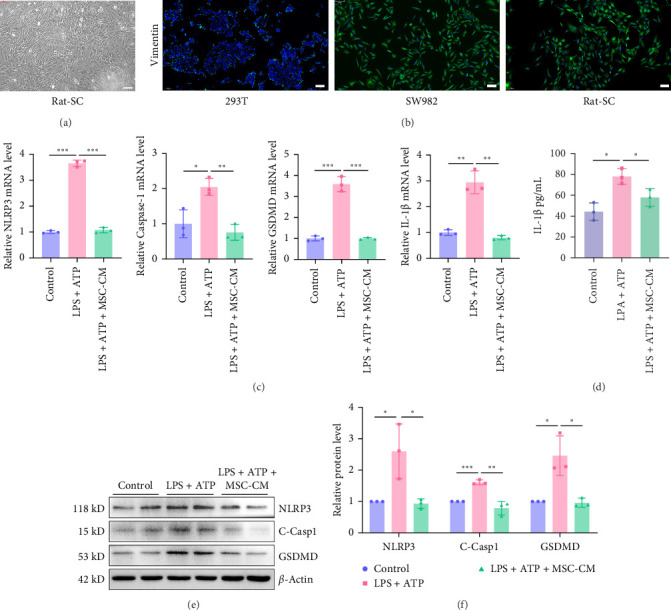
UC-MSCs inhibit NLRP3-mediated pyroptosis of primary synovial cells of rat in vitro. (A) The morphological map of primary synovial cells of rat. (B) Human embryonic kidney cells (293T), human synovial sarcoma cells (SW982), and Rat primary synovial fibroblasts (RAT-SC) were stained with vimentin by immunofluorescence. (C) qRT-PCR results of NLRP3, Caspase-1, GSDMD, and IL-1β. LPS: lipopolysaccharide, ATP: adenine nucleoside triphosphate. (*n* = 3 for each group). (D) ELISA results of IL-1β. (*n* = 3 for each group). (E) Western blot results of synovial cells NLRP3, C-Casp1, and GSDMD. (F) Image*J* software analyzed the statistics of western blot results. (*n* = 3 for each group). Data are presented as mean ± SD of individuals included in each group. *⁣*^*∗*^*p* < 0.05, *⁣*^*∗∗*^*p* < 0.01, *⁣*^*∗∗∗*^*p* < 0.001.

In order to directly illustrate the efficacy of UC-MSCs on NLRP3-mediated pyroptosis of synovial cells, we used UC-MSCs culture medium to treat the primary synovial cells pyroptosis model induced by LPS and ATP. Rat knee joint synovial cells were extracted by trypsin and collagenase digestion. The isolated rat synovial cells were spherical, suspended, and highly refractive (Figure S6). After 48 h of culture in a cell culture flask, the cells adhered to the wall. After 3 days of culture, the cells were observed under an inverted microscope to be flat, multiprocessed spindle or star-shaped (Figure 6A). Immunofluorescence staining was used, with human embryonic kidney cells (293T) as a negative control and human synovial sarcoma cells (SW982) as a positive control (Figure 6B). The results showed that Rat-scs were positive for vimentin, indicating that the cells used in this experiment met the identification criteria for rat synovial cells.

We used LPS and ATP to jointly stimulate synovial cells to undergo pyroptosis, and then treated them with the cell supernatant of UC-MSCs for 24 h. Next, qPCR method was used to detect the mRNA expression levels of pyroptosis signaling proteins NLRP3, Caspase-1, GSDMD, and IL-1β in synovial cells. The results showed (Figure 6C) that the expression levels of NLRP3, Caspase-1, GSDMD, and IL-1β mRNA in the model group were significantly higher than those in the control group, and the expression levels were significantly reduced after treatment. The experimental results showed that the synovial cell pyroptosis model was successfully constructed at the mRNA level, and UC-MSCs had a significant effect on synovial cell pyroptosis at the mRNA level.

Then the cell proteins and supernatant were extracted. Western blot and ELISA experiments were used to detect the expression levels of NLRP3, Cleaved-Caspase1, and GSDMD proteins in the cells and the expression level of IL-1β in the supernatant. The results showed (Figure 6) that compared with the control group, the protein expression levels of NLRP3, Caspase-1, GSDMD, and IL-1β in the model group were significantly increased. Compared with the model group, the protein expression levels of NLRP3, Cleaved-Caspase1, GSDMD, and IL-1β in the supernatant of the UC-MSCs treatment group were significantly reduced. Experimental results showed that a synovial cell pyroptosis model was successfully constructed at the protein level, and UC-MSCs had a significant inhibitory effect on synovial cell pyroptosis at the protein level.

The above experiments indicate that UC-MSCs can inhibit NLRP3-mediated synovial cell pyroptosis, and also provide evidence that UC-MSCs can inhibit NLRP3-mediated synovial inflammation in the early stages of OA rats in vivo.

## 4. Discussion

In recent years, although MSCs have been increasingly used in the treatment of OA, such as studies on the use of MSCs derived from bone marrow, fat or umbilical cord to treat OA patients [33–38], these studies have focused on exploring the efficacy of MSCs in the treatment of OA patients and the dosage and safety of stem cell therapy. A recent meta-analysis [39] systematically searched the Web of Science, PubMed, EMBASE, and Scopus databases, analyzing the efficacy of MSCs from various sources and at different doses across 52 studies. The results demonstrated significant improvements in both the visual analog scale (VAS) and the Knee Injury and Osteoarthritis Outcome Score (KOOS, including symptoms, pain, activities of daily living, sports, and quality of life) in the MSC group at 6-month and 12-month follow-ups. No statistically significant difference was observed in the incidence of adverse events between the MSC group and the control group, indicating that intra-articular injection of MSCs alone is safe. The study suggests that MSCs hold promise as an effective treatment for OA, though future large-sample, multicenter randomized controlled trials are needed to determine the optimal cell source and therapeutic dosage of MSCs. Overall, substantial research has been conducted on the long-term effects and safety of MSCs. However, research on the differences in the therapeutic efficacy of MSCs at various stages of the OA pathological process remains insufficient.

Recent studies on the correlation between OA staging and MSC efficacy have demonstrated the following findings: Early-stage OA (KL grades 1–2) exhibits the most significant therapeutic benefits. Structural repair potential: smaller cartilage defects allow MSC differentiation and paracrine effects to promote type II collagen synthesis and delay cartilage degradation (clinical biopsies show increased neomatrix formation) [40, 41]. Symptomatic improvement: pain relief rates exceed 80%, with functional scores improving by over 50% (e.g., significant reductions in WOMAC pain subscales) [41, 42]. Moderate-stage OA (KL grade 3) primarily achieves symptom control with limited structural repair. Pain and function: VAS scores decrease by 30%–50%, but joint mobility restoration lags behind early-stage OA; repeated injections are required to maintain efficacy (e.g., a second dose at 6-month intervals) [42, 43]. Radiographic changes: MRI reveals reduced bone marrow edema but no significant increase in cartilage thickness, suggesting anti-inflammatory effects outweigh regenerative capacity [44]. Late-stage OA (KL grade 4) remains controversial in therapeutic outcomes. Transient symptom relief: Meta-analyses indicate pain reduction fails to meet clinically important differences [44]. No structural repair: Irreversible joint space narrowing persists, with MSCs unable to reconstruct subchondral bone; 14% of studies report no improvement [44]. Elevated risks: Adverse events such as joint swelling and pain show a risk ratio of 2.67 (vs., controls) [44]. Based on the above studies, it can be observed that the therapeutic effects of MSCs vary across different stages of OA.

It is very important to learn the pathological process of OA for the selection of treatment strategies at different treatment stages. Studies have shown that in the early stages of OA, a top-down calcification process occurs, during which spherical mineral particles are formed on the surface of the joint, and in the late stage of the disease, they are transformed into fibrotic aggregates deep in the cartilage [45]. The different pathological changes in the bones and joints at different stages of the disease indicate that the structure and function of the joints have changed significantly. If the same treatment plan is used for different stages of the disease, the efficacy may be very different. Therefore, it is very important to choose the appropriate MSCs treatment plan for different stages of the disease.

In this study, rats induced by sodium iodoacetate were used as a model to investigate the pathological changes in cartilage damage, synovial inflammation and fibrosis, and chondrocyte apoptosis in the joints of OA rats in the early, middle, and late stages of the disease. By detecting the inflammatory factor IL-1β in the synovium and cartilage, we found that synovial inflammation occurred and developed rapidly in the early stage of OA, while the development rate slowed down in the middle and late stages. Imaging studies have shown that synovial inflammation may exist in early and late OA and is related to the occurrence and progression of OA. The study indicated that synovial cells orchestrate the synthesis of molecules responsible for initiating and sustaining synovial inflammation, which subsequently leads to cartilage degradation during the progression of OA [4]. We also found that the immunohistochemistry and western blot results of synovial tissues showed that synovial inflammation existed in the early, middle, and late stages of OA. In the middle stage of OA, the cartilage damage-related cytokine MMP13 and the rate of chondrocyte apoptosis increased rapidly, which means that the rate of cartilage damage is accelerating at this time. Regarding the relationship between synovial inflammation and cartilage damage in the course of this disease, we speculate that synovial inflammation in the early stage of the disease is likely to promote the rapid growth of cartilage damage in the middle stage of the disease. Related studies have also shown that in OA, synovial fibroblasts may mediate the degradation of cartilage matrix by releasing proinflammatory factors (such as IL-1β) and bone regulatory factors (such as BMP-2) [46]. In the late stage of OA, we found a significant increase in synovial tissue COL-1. By comparing the growth rates, we also found that COL-1 had the fastest growth rate in the late pathological stage, that is, 14–21 days of the disease, which indicates the rapid development of synovial fibrosis in the late pathological stage. Studies have shown that in the ACLT, DMM, and MIA models of OA synovial tissue, COL-1, CD31, VEGF, and TGF-β were significantly increased at 14 and 28 days of the disease [47]. This shows that the synovial fibrosis process is significantly accelerated at this time, which is consistent with our research.

By injecting MSCs into OA rats in three stages of the disease, we found that the expression of IL-1β in synovial tissue can be significantly reduced in the early treatment group, and the expression level of IL-1β in cartilage also can be significantly reduced in the early and middle treatment group. Although the expression level of the cartilage damage-related factor MMP13 was significantly reduced in the late treatment group, the tissue staining results and cartilage damage scores OARSI and Pelletier showed that the cartilage damage was not improved in the late treatment group. We speculate that although UC-MSCs exerted efficacy in the late-stage treatment group, the damage caused by continued chondrocyte apoptosis during the course of OA is irreversible. Chondrocytes are responsible for synthesizing extracellular matrix in articular cartilage, and the communication between chondrocytes and surrounding tissues directly or indirectly affects the progression of OA [48]. However, in the pathological state, the balance between catabolism and anabolism is broken, and the extracellular matrix is continuously degraded under the action of degradative enzymes such as MMP13. We found that the rate of chondrocyte apoptosis accelerated in the middle of the disease process, resulting in a decrease in the number of chondrocytes in the late-stage disease group. A large reduction, which may be the reason why MMP13 in cartilage tissue was significantly reduced in the late treatment group, but there was no significant effect on cartilage damage.

Recent studies have demonstrated that EVs derived from UC-MSCs can directly bind to the 3' untranslated region of NLRP3 mRNA via miR-223, thereby inhibiting chondrocyte pyroptosis in OA [21]. This finding provides compelling evidence that MSCs play a significant role in mitigating chondrocyte pyroptosis in OA. Studies have also found that Chrysin can improve NLRP3-mediated synovial inflammation in OA [49], which indicates that OA can be improved by inhibiting NLRP3-mediated synovial inflammation. This suggests that MSCs may improve OA by inhibiting NLRP3-mediated synovial inflammation. We found that NLRP3-related pyroptosis protein in the synovium began to be significantly expressed in the early stages of OA rats, which is consistent with other studies [4]. In vivo, through immunohistochemistry of synovial tissue of OA rats, we found that the expression of synovial pyroptosis-related proteins NLRP3, GSDMD, and IL-1β was significantly reduced in the early treatment group. Studies have found that LPS and ATP can induce and initiate NLRP3-mediated pyroptosis [50, 51]. In vitro, we used LPS and ATP to induce pyroptosis in Rat-scs. We found that the expressions of NLRP3, GSDMD, Caspase-1, and IL-1β were significantly increased at the mRNA and protein levels, and after treatment with UC-MSCs supernatant, the expressions were significantly decreased. This demonstrates the effectiveness of UC-MSCs in inhibiting pyroptosis of rat synovial cells. Indicators related to NLRP3 in the synovium of OA rats and cartilage damage showed a positive correlation at different treatment time points, which also showed that UC-MSCs slowed down the pathological process of OA rats by inhibiting NLRP3-mediated synovial cell pyroptosis.

The regulatory mechanism of MSCs on NLRP3-mediated cell pyroptosis is constantly being discovered. Studies have shown that EVs derived from UC-MSCs reduce NLRP3 m6A in macrophages by interacting with METTL3, thereby, alleviating OA in mice [52]. Human adipose-derived MSCs attenuate OA by inhibiting chondrocyte pyroptosis [53]. Bone marrow MSCs pretreated with LPS can regulate macrophage polarization and inflammation through the NF-κB/NLRP3/procaspase-1/IL-1β signaling pathway and improve immunosuppression in allogeneic skin transplantation [54]. Exosomes from UC-MSCs prevent ischemic muscle damage by releasing circHIPK3 to inhibit cell pyroptosis, and can inhibit NLRP3-mediated pyroptosis in C2C12 cells stimulated by LPS and ATP in vitro [55]. Stem cell-derived exosomes repair ischemic muscle damage by inhibiting the NLRP3 inflammasome pathway mediated by the tumor suppressor factor Rb1 [56]. These studies have shown that proteins, miRNAs, and circRNAs secreted by MSCs and their exosomes can regulate NLRP3-mediated pyroptosis in immune and inflammatory diseases, thereby, improving the pathological progression of the disease. The early stages of the disease are the best time to treat the disease. Our study also showed that UC-MSCs has the best effect on cartilage damage in the early stages of OA rats, so the choice of treatment strategy for UC-MSCs in the early stages of OA is particularly important. Our study clarified that the rapid development of NLRP3-mediated synovial inflammation in the early stages of OA rats promoted the progression of OA in the middle and late stages of the disease, and UC-MSCs only had a significant effect on NLRP3-mediated synovial inflammation in the early stages of the disease.

## 5. Conclusions

In this study, sodium iodoacetate-induced OA rats were used as a model to explore the early stage of OA rats, which was the main development stage of NLRP3-mediated synovial inflammation; the middle stage of OA rats was the main development stage of chondrocyte apoptosis; and the late stage of OA rats was the main development stage of synovial fibrosis. UC-MSCs can reduce cartilage damage and chondrocyte apoptosis in the early and middle stages of OA rats. In addition, UC-MSCs improved synovial inflammation by inhibiting NLRP3-mediated synovial cells pyroptosis in the early stages of OA rats, thereby, effectively reducing cartilage damage in OA. In summary, our study provides a direction and theoretical experimental basis for the selection of strategies for UC-MSCs to treat knee OA. UC-MSCs inhibiting NLRP3-mediated synovial inflammation in the early stages of OA can be used as an important therapeutic strategy for UC-MSCs for OA.

## Data Availability

All the data generated and/or analyzed during this study are included in this published article.

## Supporting Information

Supporting Information ARRIVE checklist enumerates the 10 fundamental principles adhered to in animal experimentation. Figure S1. Schematic diagram of animal experimental design. Figure S2. Negative control for MSC characterization. Figure S3. Characterization of MSCs in DMEM and serum-free medium. Figure S4. Original western blot bands of rat pathology. Figure S5. Original western blot bands of rat efficacy. Figure S6. Nonadherent rat synovial cells. Table S1. Primer sequences for qRT-PCR. Table S2. Reverse transcription system. Table S3. qPCR system. Table S4. qPCR program settings. Table S5. RNA quality parameters.
