# Supplementary material for: Human Umbilical Cord Mesenchymal Stem Cells Inhibit the Progression of Osteoarthritis by Suppressing NLRP3-Mediated Synovial Inflammation in the Early Stages of the Disease
Source: Stem Cells Int. 2025 Aug 30;2025:7558817. doi: 10.1155/sci/7558817 (PMC12413943; doi:10.1155/sci/7558817)
Supplement: Supporting Information — ARRIVE checklist enumerates the 10 fundamental principles adhered to in animal experimentation. Figure S1. Schematic diagram of animal experimental design. Figure S2. Negative control for MSC characterization. Figure S3. Characterization of MSCs in DMEM and serum-free medium. Figure S4. Original western blot bands of rat pathology. Figure S5. Original western blot bands of rat efficacy. Figure S6. Nonadherent rat synovial cells. Table S1. Primer sequences for qRT-PCR. Table S2. Reverse transcription system. Table S3. qPCR system. Table S4. qPCR program settings. Table S5. RNA quality parameters. [file 7558817.f1.zip › Supplementary Material/Supplement Figure legends and Tables.docx]

**Supplementary Material**

**ARRIVE checklist.**

This table enumerates the ten fundamental principles adhered to in animal experimentation.

Study design: (a) Figure S1. (b) Each animal serves as an experimental unit.

Sample size: (a) The experimental animals were divided into 7 groups with 5 animals each, totaling 35 rats. (b) The number of animals per group was determined based on previous similar studies[1].

Inclusion and exclusion criteria: (a) If any animals died, they would be excluded from the study. (b) All experimental animals were included in the outcome statistics. The selection of treatment and modeling time points was based on existing studies[2] with a sample size of 5 for each experimental group. (c) Each experimental group consisted of 5 rats (n=5).

Randomisation: (a) All animals were randomly divided into 7 groups (5 rats/group): control group, OA-1W group, OA-2W group, OA-3W group, MSC-Early group, MSC-Mid group, and MSC-Late group. Random numbers were generated using the standard =RAND () function in Microsoft Excel. (b) One cage per group, with seven cages randomly assigned to two levels on the rack using a function, and the tier positions were exchanged once daily. The experimental sequence for each animal within each group was randomly generated once per session by the function.

Blinding: Two distinct researchers were involved in the animal experiments, as follows: One researcher, who was blinded to the allocation of experimental animals, was responsible for administering the modeling drugs and cellular therapeutics, collecting animal outcomes, and subsequently recording the allocation details. The other researcher, who was blinded to both group assignments and experimental results, performed the data analysis.

Outcome measures: (a) The following parameters were evaluated: cartilage wear status (Pelletier score, OARSI score), chondrocyte apoptosis rate (TUNEL), inflammatory factors (IL-1β), fibrosis factors (COL-1), matrix metalloproteinases (MMP13), and pyroptosis-related factors(NLRP3, GSDMD). (b) The primary outcome measures of this study were indicators related to cartilage wear (Pelletier score, OARSI score) in rats and inflammasome-associated (NLRP3, IL-1β ) parameters, while the secondary outcomes included fibrosis (COL-1) and chondrocyte apoptosis results (TUNEL).

Statistical methods: (a) All experiments in this study were performed in triplicate or more. The presented data represent mean values ± standard deviation, and statistical analysis was conducted using GraphPad Prism 8.0.2. Data were first assessed for normality using the Shapiro-Wilk test, followed by evaluation of variance homogeneity with the Brown-Forsythe test. For data conforming to both normality and homoscedasticity, one-way ANOVA was employed, followed by Tukey's multiple comparison test. In cases of unequal variances, Welch's ANOVA correction was applied. A p-value < 0.05 was considered statistically significant. (b) The methods for evaluating the data conform to all the assumptions of the hypothesis.

Experimental animals: (a) Purchase male Sprague-Dawley rats aged 6 weeks with body weights ranging from 200-250 g from the Bestcell Model Biological Center. (b) The health status of the animals should be Specific Pathogen Free (SPF) grade.

Experimental procedures: (a) Six-week-old SD rats were purchased from the Baisai Model Organism Center and randomly divided into two groups: the osteoarthritis group (OA) (n=30) and the control group (n=5). Sodium monoiodoacetate (MIA) (1.2 g) was dissolved in 4 mL of purified water to prepare a solution. The solution was filtered through a 0.22 μm filter in a biosafety cabinet, yielding a final concentration of 1.5 mg/50 μL MIA solution. The solution was transported to the animal facility in ice water. The OA group received an intra-articular injection of MIA solution at a dose of 1.5 mg/50 μL per 200 g body weight into the knee joint cavity, while the control group received an equal volume of purified water.

(b) The day of administration was designated as Day 0. Three days later, the OA group rats were randomly subdivided into six groups: the OA-1W pathological group (OA-1W, n=5), the OA-2W pathological group (OA-2W, n=5), the OA-3W pathological group (OA-3W, n=5), the early mesenchymal stem cell treatment group (MSC-Early, n=5), the mid-term mesenchymal stem cell treatment group (MSC-Mid, n=5), and the late mesenchymal stem cell treatment group (MSC-Late, n=5).

At this time, the MSC-Early group received UC-MSC therapy, with an intra-articular injection of 2.5×10⁵ UC-MSCs suspended in 50 μL saline into the knee joint cavity. One week after Day 0 (modeling), the OA-1W group rats were euthanized via excessive isoflurane inhalation. The knee joints and partial synovial tissues were harvested. The joints were fixed in 4% paraformaldehyde for further analysis, while the synovial tissues were flash-frozen in liquid nitrogen and stored at −80°C.

One week after the MSC-Early group treatment, the MSC-Mid group received UC-MSC therapy, with an intra-articular injection of 2.5×10⁵ UC-MSCs suspended in 50 μL saline.

Two weeks after Day 0, the OA-2W group rats were euthanized via excessive isoflurane inhalation. The knee joints and partial synovial tissues were harvested and processed as described above.

Two weeks after the MSC-Early group treatment, the MSC-Late group received UC-MSC therapy, with an intra-articular injection of 2.5×10⁵ UC-MSCs suspended in 50 μL saline. Three weeks after Day 0, the control group, OA-3W group, MSC-Early group, MSC-Mid group, and MSC-Late group rats were euthanized via excessive isoflurane inhalation. The knee joints and synovial tissues were collected and processed as previously described.

(c) The rats were housed under controlled conditions with 45–55% humidity, a temperature of 20±2°C, and a 12-hour light-dark cycle, with free access to food and water. After a three-day acclimatization period, the experiments commenced.

(d) Intra-articular injection of MIA is a widely used method for chemically inducing osteoarthritis (OA) in animal models. This approach primarily inhibits key glycolytic enzymes (e.g., glyceraldehyde-3-phosphate dehydrogenase), leading to energy metabolism dysfunction and apoptosis of chondrocytes, thereby mimicking the pathological features of human OA. This method is well-established and ensures minimal interference from other factors when investigating therapeutic interventions at different stages of OA progression.

Results: (a) All data from the experimental animals have been presented in the results figures, including individual data points, mean values, error measurements, and dispersion parameters. Descriptive statistics will not be provided separately. (b) The following presents the effect sizes and confidence intervals for the key data. Figure 2G [η^2^ = 0.87; Control CI_95_ (1.15*10^-5^, 3.6*10^-3^), OA-1W CI_95_ (0.005, 0.019), OA-2W CI_95_ (0.015, 0.03), OA-3W CI_95_ (0.048, 0.107) ]; Figure 2I [η^2^ = 0.89; Control CI_95_ (1.12, 2.09), OA-1W CI_95_ (4.64, 8.06), OA-2W CI_95_ (11.32, 17.61), OA-3W CI_95_ (12.36, 21.63) ]; Figure 3D [η^2^ = 0.55; Control CI_95_ (-0.00085, 0.007), OA-1W CI_95_ (0.022, 0.1), OA-2W CI_95_ (0.033, 0.11), OA-3W CI_95_ (0.027, 0.12) ]; Figure 4G [η^2^ = 0.82; Control CI_95_ (1.15*10^-5^, 0.0036), OA CI_95_ (0.048, 0.11), MSC-Early CI_95_ (0.023, 0.045), MSC-Mid CI_95_ (0.03, 0.084), MSC-Late CI_95_ (0.077, 0.13) ]; Figure 4I [η^2^ = 0.89; Control CI_95_ (1.12, 2.1), OA CI_95_ (0.048, 0.11), MSC-Early CI_95_ (2.25, 4.92), MSC-Mid CI_95_ (5.17, 8.9), MSC-Late CI_95_ (9.55, 16.28) ]; Figure 5B [η^2^ = 0.87; Control CI_95_ (0.0021, 0.01), OA CI_95_ (0.042, 0.075), MSC-Early CI_95_ (0.0068, 0.034), MSC-Mid CI_95_ (0.038, 0.068), MSC-Late CI_95_ (0.058, 0.069) ]; Figure 5D [η^2^ = 0.81; Control CI_95_ (-0.00085, 0.0074), OA CI_95_ (0.027, 0.12), MSC-Early CI_95_ (0.0032, 0.0087), MSC-Mid CI_95_ (0.02, 0.072), MSC-Late CI_95_ (0.046, 0.074) ].

**Figure S1. Schematic diagram of animal experimental design.**

On dayular injection of 2.5×10^5^ UC-MSCs. Finally, on day 21, specimens were collected from the control group, MSC-Early group, MSC-Mid group, MSC-Late group, and OA-3W group.

**Figure S2. Negative control for MSC characterization.**

Negative control for MSC markers (CD90^+^, CD105^+^, CD73^+^, CD34^-^，CD19^-^, CD45^-^, HLA-DR^-^).

**Figure S3. Characterization of MSCs in DMEM and Serum-Free Medium**

Comparison of MSC markers (CD90^+^, CD105^+^, CD73^+^, CD34^-^，CD19^-^, CD45^-^, HLA-DR^-^) between groups DMEM and Serum-Free Medium.

**Figure S4. Original Western blot bands of rat pathology**

Original Western blot bands of the pathological progression in rat synovial tissue.

**Figure S5. Original Western blot bands of rat efficacy**

Original Western blot bands of rat synovial tissue for efficacy assessment.

**Figure S6. Non-adherent rat synovial cells**

Morphology of rat synovial cells at the time of inoculation. Scale bar: 100 μm. Figure (B) is a twofold magnification of Figure (A) .

**Table S1. Primer sequences for qRT-PCR**

| **Gene** | **Genus** | **Forword primer (5’-3’)** | **Reverse primer (5’-3’)** |
| --- | --- | --- | --- |
| NLRP3 | Rat | CAGACCTCCAAGACCACGACTG | CATCCGCAGCCAATGAACAGAG |
| GSDMD | Rat | CACGGGACAAGGGAAAATTTC | AGGATTTTGTTTTCAGGCTGC |
| Caspase1 | Rat | CACATGAAAGAATATGCCTGGTC | GTCCTGGGAAGAGGTAGAAAC |
| IL-1β | Rat | CTTGACTTGGGCTGTCCAGA | ACGGGCAAGACATAGGTAGC |
| β-actin | Rat | TGCCCATCTATGAGGGTTAC | CTCCAGGGAGGAAGAGGAT |

**Table S2. Reverse transcription system**

| **Reagent** | **Volume** |
| --- | --- |
| RNA Sample | 1 μg |
| gDNA Remover | 1 μL |
| 5×TransScript® Uni All-in-One SuperMix for qPCR | 4 μL |
| DEPC Water | Supplement to 20 μL |

The dosage of each reagent in reverse transcription.

**Table S3. qPCR** **system**

| **Reagent** | **Volume** |
| --- | --- |
| Forward Primer (10 μM) | 0.5 μL |
| 2×PerfectStart® Green qPCR SuperMix | 5 μL |
| Reverse Primer (10 μM) | 0.5 μL |
| DEPC Water | 3 μL |
| cDNA | 1 μL |

The dosage of each reagent in qPCR.

**Table S4. qPCR** **program settings**

| **Procedure** | **Temperature** | **Duration** |
| --- | --- | --- |
| Initial Denaturation | 93℃ | 35 s |
| Denaturation | 95℃ | 6 s |
| Annealing | 61℃ | 16 s |
| Extension | 73℃ | 11 s |
| Cycle 42 times  1 μL | | |

The procedural steps and time allocation in qPCR.

**Table S5. RNA quality parameters**

| **Group** | **A260/A230** | **A260/A280** | **Concentration（ng/μL）** |
| --- | --- | --- | --- |
| H_2_O | 0.7 | 0.53 | -0.13 |
| Control 1 | 2.07 | 2.07 | 1089.66 |
| Model 1 | 2.04 | 2.07 | 1003.38 |
| Treatment 1 | 1.41 | 2.01 | 1257.02 |
| Control 2 | 2.12 | 2.07 | 881.99 |
| Model 2 | 1.98 | 2.07 | 810.47 |
| Treatment 2 | 1.41 | 2.02 | 1023.66 |
| Control 3 | 2.12 | 2.05 | 677.38 |
| Model 3 | 1.96 | 2.06 | 696.91 |
| Treatment 3 | 1.38 | 2.02 | 695.84 |

**References**

[1] Lei Y, Wang Y, Shen J, et al., *Injectable hydrogel microspheres with self-renewable hydration layers alleviate osteoarthritis.* Sci Adv, 2022. 8(5): p. eabl6449.

[2] He L, He T, Xing J, et al., *Bone marrow mesenchymal stem cell-derived exosomes protect cartilage damage and relieve knee osteoarthritis pain in a rat model of osteoarthritis.* Stem Cell Res Ther, 2020. 11(1): p. 276.
