# Supplementary figures and images for: Human Umbilical Cord Mesenchymal Stem Cells Inhibit the Progression of Osteoarthritis by Suppressing NLRP3-Mediated Synovial Inflammation in the Early Stages of the Disease
Source: Stem Cells Int. 2025 Aug 30;2025:7558817. doi: 10.1155/sci/7558817 (PMC12413943; doi:10.1155/sci/7558817)

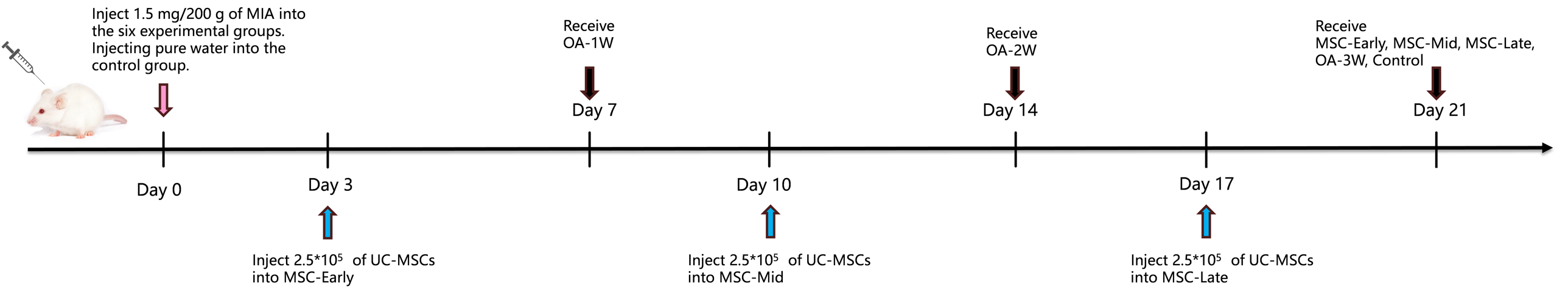

Supplement: Supporting Information — ARRIVE checklist enumerates the 10 fundamental principles adhered to in animal experimentation. Figure S1. Schematic diagram of animal experimental design. Figure S2. Negative control for MSC characterization. Figure S3. Characterization of MSCs in DMEM and serum-free medium. Figure S4. Original western blot bands of rat pathology. Figure S5. Original western blot bands of rat efficacy. Figure S6. Nonadherent rat synovial cells. Table S1. Primer sequences for qRT-PCR. Table S2. Reverse transcription system. Table S3. qPCR system. Table S4. qPCR program settings. Table S5. RNA quality parameters. [file 7558817.f1.zip › Supplementary Material/Figure S1.tif]

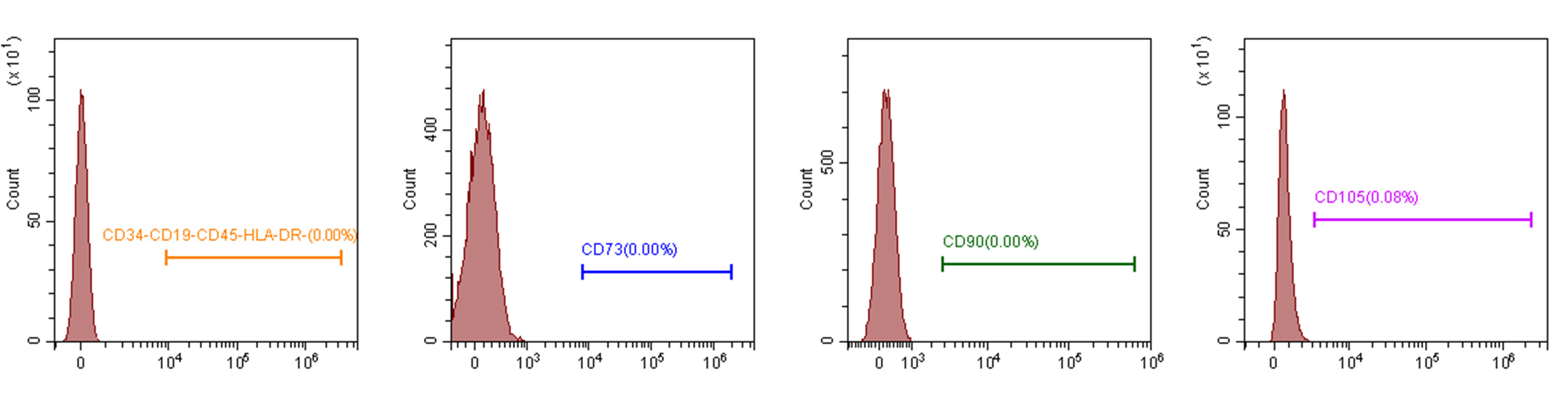

Supplement: Supporting Information — ARRIVE checklist enumerates the 10 fundamental principles adhered to in animal experimentation. Figure S1. Schematic diagram of animal experimental design. Figure S2. Negative control for MSC characterization. Figure S3. Characterization of MSCs in DMEM and serum-free medium. Figure S4. Original western blot bands of rat pathology. Figure S5. Original western blot bands of rat efficacy. Figure S6. Nonadherent rat synovial cells. Table S1. Primer sequences for qRT-PCR. Table S2. Reverse transcription system. Table S3. qPCR system. Table S4. qPCR program settings. Table S5. RNA quality parameters. [file 7558817.f1.zip › Supplementary Material/Figure S2.tif]

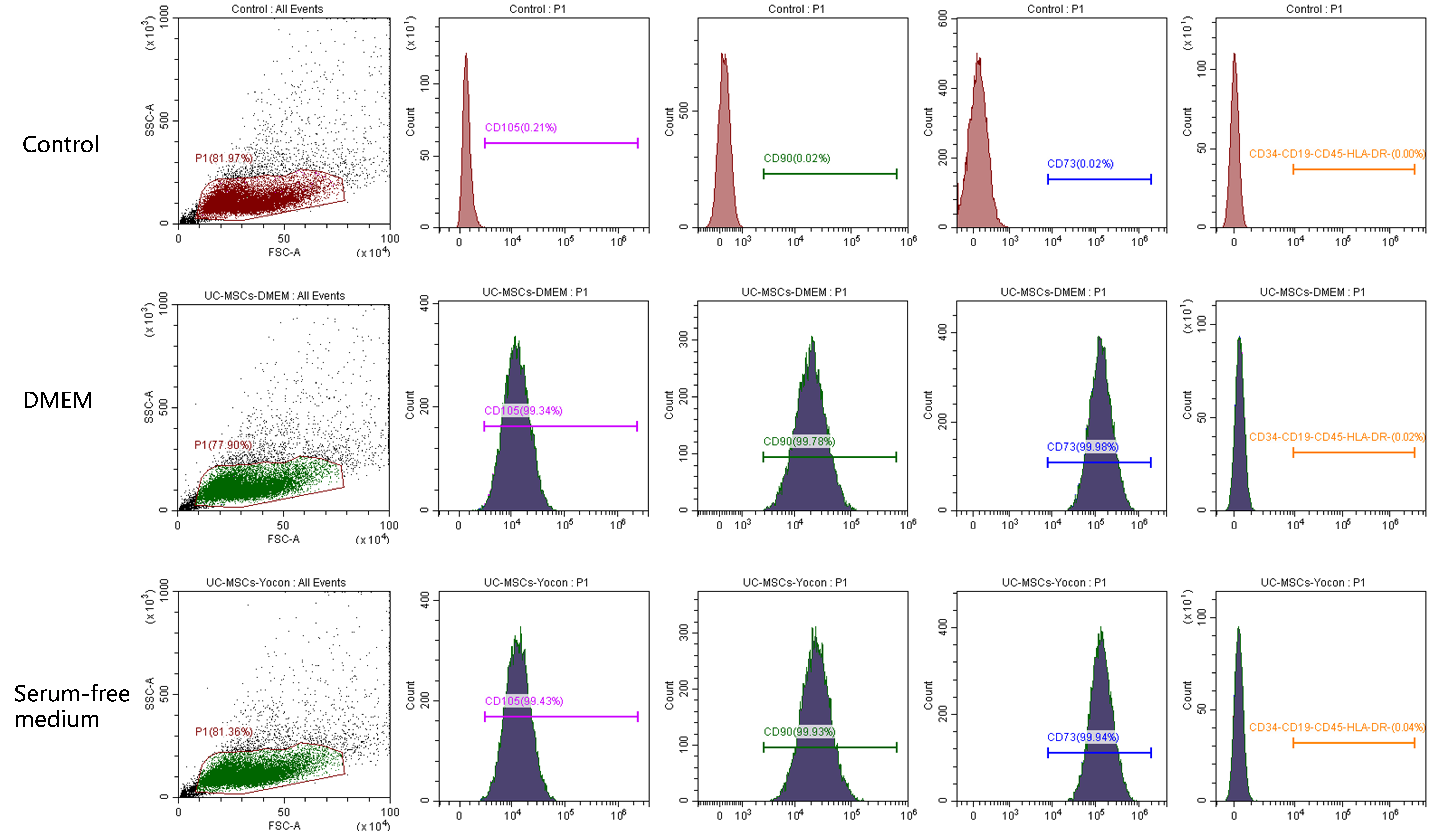

Supplement: Supporting Information — ARRIVE checklist enumerates the 10 fundamental principles adhered to in animal experimentation. Figure S1. Schematic diagram of animal experimental design. Figure S2. Negative control for MSC characterization. Figure S3. Characterization of MSCs in DMEM and serum-free medium. Figure S4. Original western blot bands of rat pathology. Figure S5. Original western blot bands of rat efficacy. Figure S6. Nonadherent rat synovial cells. Table S1. Primer sequences for qRT-PCR. Table S2. Reverse transcription system. Table S3. qPCR system. Table S4. qPCR program settings. Table S5. RNA quality parameters. [file 7558817.f1.zip › Supplementary Material/Figure S3.tif]

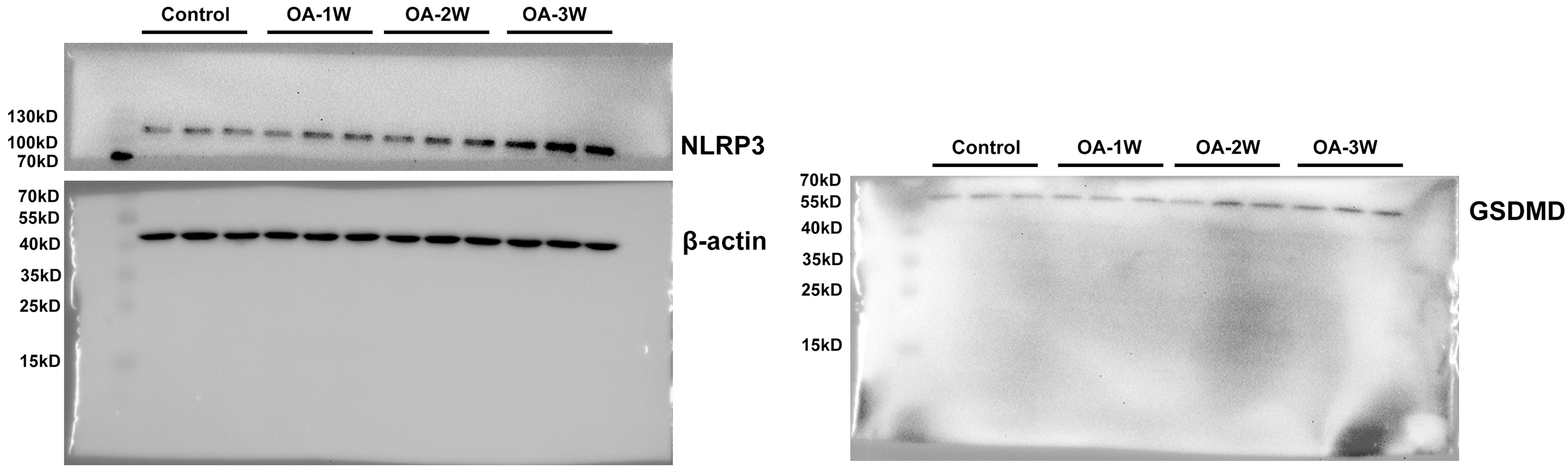

Supplement: Supporting Information — ARRIVE checklist enumerates the 10 fundamental principles adhered to in animal experimentation. Figure S1. Schematic diagram of animal experimental design. Figure S2. Negative control for MSC characterization. Figure S3. Characterization of MSCs in DMEM and serum-free medium. Figure S4. Original western blot bands of rat pathology. Figure S5. Original western blot bands of rat efficacy. Figure S6. Nonadherent rat synovial cells. Table S1. Primer sequences for qRT-PCR. Table S2. Reverse transcription system. Table S3. qPCR system. Table S4. qPCR program settings. Table S5. RNA quality parameters. [file 7558817.f1.zip › Supplementary Material/Figure S4.tif]

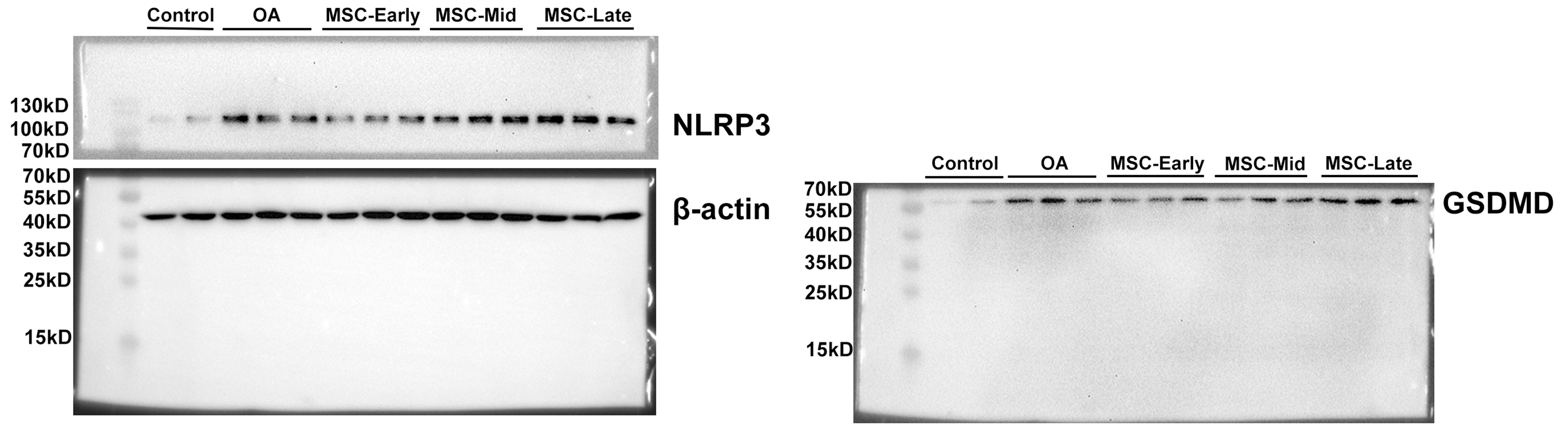

Supplement: Supporting Information — ARRIVE checklist enumerates the 10 fundamental principles adhered to in animal experimentation. Figure S1. Schematic diagram of animal experimental design. Figure S2. Negative control for MSC characterization. Figure S3. Characterization of MSCs in DMEM and serum-free medium. Figure S4. Original western blot bands of rat pathology. Figure S5. Original western blot bands of rat efficacy. Figure S6. Nonadherent rat synovial cells. Table S1. Primer sequences for qRT-PCR. Table S2. Reverse transcription system. Table S3. qPCR system. Table S4. qPCR program settings. Table S5. RNA quality parameters. [file 7558817.f1.zip › Supplementary Material/Figure S5.tif]

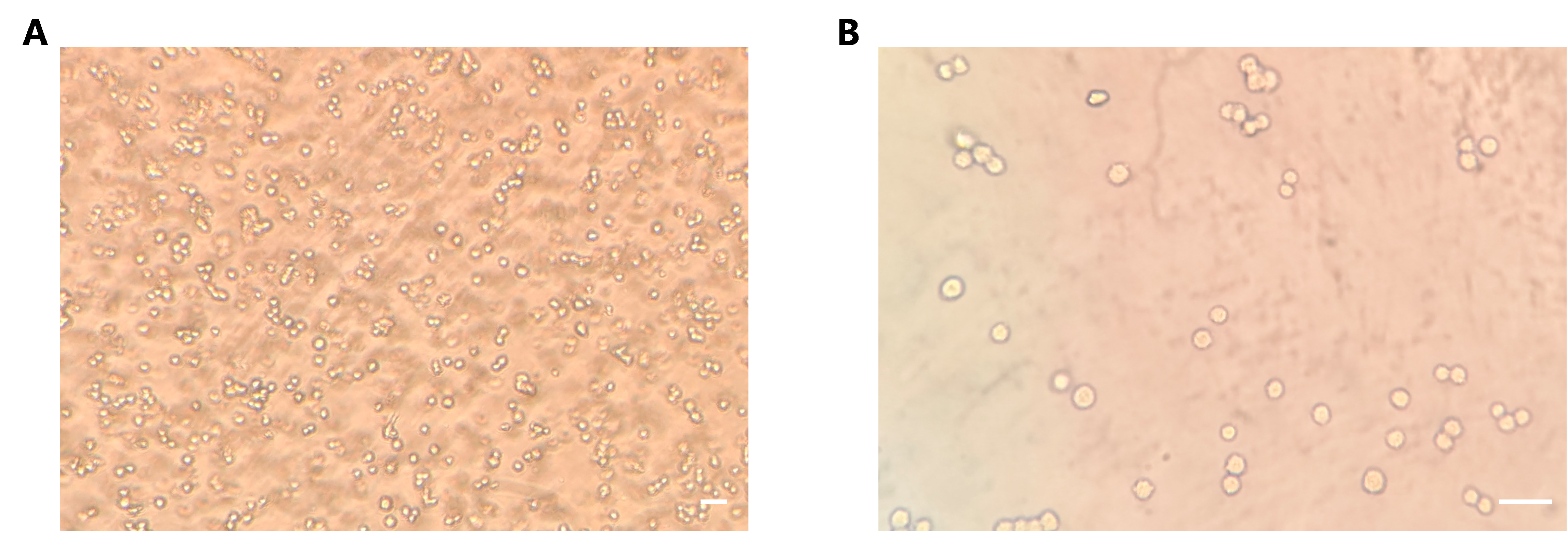

Supplement: Supporting Information — ARRIVE checklist enumerates the 10 fundamental principles adhered to in animal experimentation. Figure S1. Schematic diagram of animal experimental design. Figure S2. Negative control for MSC characterization. Figure S3. Characterization of MSCs in DMEM and serum-free medium. Figure S4. Original western blot bands of rat pathology. Figure S5. Original western blot bands of rat efficacy. Figure S6. Nonadherent rat synovial cells. Table S1. Primer sequences for qRT-PCR. Table S2. Reverse transcription system. Table S3. qPCR system. Table S4. qPCR program settings. Table S5. RNA quality parameters. [file 7558817.f1.zip › Supplementary Material/Figure S6.tif]
